# Mr. Bellamy, on Catarrhal Fever

**Published:** 1800-10

**Authors:** G. Bellamy

**Affiliations:** Surgeon. Spencer, Portsmouth Harbour


### *To the Editors of the Medical and Phyſical Journal.*

GENTLEMEN,

THE ſucceſsful treatment of infectious Catarrhal Fever muſt be intereſting to ſociety at large; and more eſpecially perhaps in naval and military ſervice, wherein there are ſo many occaſional cauſes occuring to its production. I am,

GENTLEMEN,

Your very humble ſervant,

G. BELLAMY, Surgeon.

*Spencer, Portſmouth Harbour,*

*July* 26, 1800.

---

An infectious Catarrhal Fever which exiſted with different degrees of violence on board His Majeſty's ſhip Bellerophon, from the beginning of March to the end of April, 1797, I believe to have ariſen from the following cauſes:

1ſt. The ſeaſon of the year, which was particularly moiſt with raw coldneſs, and a bleak eaſterly wind, while we were fitting fitting at Spithead for foreign fervice; fhortly after which the difeafe took place.

2dly. The fatigue of the people in a hafty equipment, and the confiderable addition of various ftores, by which the fhip was much crowded, preventing a free circulation of air, and affifting the ill influence of that from the atmofphere; which, whether it brought a difeafe *sui generis*, diftinct in its form, and immediately acting on the body, predifpofed for its reception; at leaft, acted as a debilitating caufe, whereby the body became fubject to fever, and marked by thofe fecondary fymptoms, of affections of parts of the body moft eafily influenced by the occafional caufe, i. e. cold and moifture. Indeed, I rather think that at all times, though the catarrh be the prominent feature, that it is not the effence of the difeafe; and that it fhould rather be confidered as fever with catarrh; that the former fhould be the object of treatment, and that the pulmonic and other fymptoms fhould only be confidered as of a fecondary nature; that they fhould not contradict the treatment of the fever, only when they become violent, and point out the neceffity of fome fudden relief for the prevention of abfcefs of the lungs.

This was the almoft uniform train of fymptoms: The patients came in the morning, feveral together; complained of being taken ill in the night, fome few of having gone to bed rather unwell; but always the alteration from health to ficknefs was fudden. Languor and defpondency, naufea, fometimes vomiting; a fenfe of cold and exhauftion, (nothing like rigors fucceeded by ftrong febrile heat); great pain of the head and loins; giddinefs, almoft drawing their limbs after them; oblig-ed to be fupported in the more fevere cafes; hollownefs of the eyes, with blacknefs of the eye-lids; fœtid breath, fhort cough, and pain of the cheft; pulfe quick and fmall, tongue whitifh and trembling. When the fever was abated, or gone off, there was a long continuance of cough and debility; and in a few, the induction of chronic diftempers; one of dropfy; the difpofition to typhus was too plain. The indications of cure appeared to be:—Firft, The removal, or correction of the oftenfible caufes; for which purpofe the different parts of the fhip, wherever bad air might be confined, or generated, were emptied, fumigated with the nitrous gas; wafhed, dried by fires, and then wafhed with lime and vinegar. The lower gun-deck was alfo fumigated, as directed, with the nitrous gas, whilft the men were in their hammocks; and thefe meafures were often repeated. The fick birth, and bedding of the fick, daily the fame, unlefs when prevented by the violence of thofe pulmonic fymptoms which the gas always provoked. The fick were, were, as much as poſſible, ſeparated from the ſhip's company, and every care uſed that none but the nurſes ſhould attend them. And here is the ſtrongeſt aſſurance of the infectious nature of the fever; for when all theſe occaſional cauſes had been removed, and when even we had changed our climate by going to Liſbon, the number of ſick did not leſſen, though the violence of the diſeaſe certainly abated; therefore, the permanence of the diſeaſe, and the number of men who ſucceſſively fell ill of it, muſt have been from contagion. The perſons of the ſick were alſo kept as clean as poſſible, and thoſe who were confined to bed were put apart.

Secondly, After having evacuated the primæ viæ by a gentle emetic, and procured a ſtool or two, which removed ſo much offending matter, I gave, every ſix hours, four ounces of wine and one of lemon juice, as a cordial antiputreſcent draught, and plenty of a pectoral decoction, which was alſo well acidulated. Where the oppreſſion of the cheſt was violent a pediluvium; and at night promoted gentle perſpiration by antimony and opium. In ſtill more urgent caſes, either of debility or pectoral affection, repeated bliſters between the ſhoulders, and on the breaſt, from which immediate relief was experienced. When the pectoral complaints were abated, and debility conſiderable, tonics ſeemed to be indicated, but did not anſwer, as they always brought on pain of the breaſt, &c. with increaſed violence; and as I found the above draught very efficacious, I forbore the bark, &c. till a ſtate of thorough convaleſcence, which was aſſiſted by an addition of wine, gentle exerciſe, and regulating the bowels.

With any other diſpoſition of fever venæſection would have been proper, where pulmonic inflammation was dreaded. In the caſe of thoſe who remained long affected with cough, &c. when the fever was entirely gone, bliſters and pectoral drinks were relied on, and proved equal to expectation, for I had the ſatisfaction not to loſe a man.

